# Effects of Environmental Conditions on Athlete’s Cardiovascular System

**DOI:** 10.3390/jcm13164961

**Published:** 2024-08-22

**Authors:** Andrea Segreti, Mihail Celeski, Emiliano Guerra, Simone Pasquale Crispino, Francesca Vespasiano, Lorenzo Buzzelli, Chiara Fossati, Rocco Papalia, Fabio Pigozzi, Francesco Grigioni

**Affiliations:** 1 Cardiology Unit, Fondazione Policlinico Universitario Campus Bio-Medico, Via Alvaro del Portillo, 200, 00128 Roma, Italy; mihail.celeski@unicampus.it (M.C.); simone.crispino@unicampus.it (S.P.C.); francesca.vespasiano@unicampus.it (F.V.); lorenzo.buzzelli@unicampus.it (L.B.); f.grigioni@policlinicocampus.it (F.G.); 2 Research Unit of Cardiovascular Science, Department of Medicine and Surgery, Università Campus Bio-Medico di Roma, Via Alvaro del Portillo, 21, 00128 Roma, Italy; 3 Department of Movement, Human and Health Sciences, University of Rome “Foro Italico”, Piazza Lauro de Bosis, 15, 00135 Roma, Italy; chiara.fossati@uniroma4.it (C.F.); fabio.pigozzi@uniroma4.it (F.P.); 4 Cardiology Division, Department of Biomedical, Metabolic and Neural Sciences, University of Modena and Reggio Emilia, Policlinico di Modena, Via del Pozzo, 71, 41124 Modena, Italy; emilianoguerra27@gmail.com; 5 Department of Orthopaedic and Trauma Surgery, Università Campus Bio-Medico di Roma, Via Alvaro del Portillo, 21, 00128 Roma, Italy; r.papalia@policlinicocampus.it; 6 Research Unit of Orthopaedic and Trauma Surgery, Fondazione Policlinico Universitario Campus Bio-Medico, Via Alvaro del Portillo, 200, 00128 Roma, Italy

**Keywords:** adaptation, athletes, environmental conditions, cardiovascular efficiency

## Abstract

Environmental factors such as extreme temperatures, humidity, wind, pollution, altitude, and diving can significantly impact athletes’ cardiovascular systems, potentially hindering their performance, particularly in outdoor sports. The urgency of this issue is heightened by the increasing prevalence of climate change and its associated conditions, including fluctuating pollution levels, temperature variations, and the spread of infectious diseases. Despite its critical importance, this topic is often overlooked in sports medicine. This narrative review seeks to address this gap by providing a comprehensive, evidence-based evaluation of how athletes respond to environmental stresses. A thorough assessment of current knowledge is essential to better prepare athletes for competition under environmental stress and to minimize the harmful effects of these factors. Specifically, adaptative strategies and preventative measures are vital to mitigating these environmental influences and ensuring athletes’ safety.

## 1. Introduction

Research consistently demonstrates a positive relationship between exposure to natural and green environments and overall health [1,2]. Natural settings have been shown to alleviate stress, accelerate recovery from stressful situations, enhance cognitive function, and improve cardiovascular efficiency [1,2]. Training in outdoor environments, as opposed to indoor settings, not only enhances performance but also boosts well-being indicators [1,2]. The increasing accessibility of natural spaces and improved infrastructure has created exciting opportunities for millions of travelers to engage in outdoor leisure activities and participate in competitive events, often held in challenging conditions [3,4]. As a result, various environmental factors, particularly extreme weather conditions such as cold air, rain, extreme heat or warm or cold, pollution, high altitude, or deep-sea environments, can influence outdoor sports performance [5,6,7,8,9].

## 2. Effects of Environmental Factors

**Figure 1:**
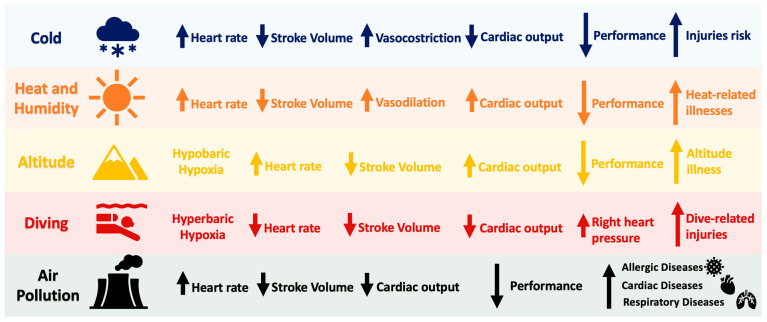
Acute effects of varying environmental conditions on the cardiovascular system and athletic performance. Notably, the chronic response of various cardiovascular parameters after adaptation mechanisms differs from that of the acute response depicted in the figure. ↑ = increase; ↓ = reduction.

Figure 1 illustrates the unique and consistent effects of various environmental conditions on athletes’ health and performance.

Humans possess a range of adaptive biological mechanisms and behavioral responses that allow them to cope with environmental stressors [10]. Typically, adaptation occurs after repeated exposure to specific stressors [11,12]. However, the adaptation mechanisms differ between “normal” athletes and those with concomitant cardiomyopathy. Additionally, individuals with pre-existing medical conditions may face a significantly increased risk of cardiovascular adverse events when exposed to certain stressors [11]. In this context, environmental factors can contribute both to the mechanisms of adaptation and the induction of cardiovascular events.

Outdoor training and competitions can vary significantly in duration, ranging from a few seconds to ultra-marathons lasting over 24 h [13,14]. This variability highlights the complexity and the necessity of comprehensive research to understand how athletes across different sports develop cardiovascular mechanisms to adapt to varied environmental conditions. As illustrated in Figure 1, these mechanisms include changes in heart rate (HR), blood pressure (BP), and cardiac output (Q). These physiological responses are crucial for maintaining performance across different environmental conditions, underscoring the importance of this topic.

Finally, it is essential to consider the potential relationship between cardiac chamber diameter or volume, body surface area, and environmental adaptation mechanisms. There is substantial evidence supporting racial differences in cardiac chamber size. However, differences in left ventricular end-diastolic volume among Asian, Black, and White populations are minimal when indexed to body surface area (BSA) [15]. Racial variation in cardiac chamber size may be linked to ancestral environmental adaptation mechanisms. Nonetheless, current understanding suggests that the predominant factors influencing cardiac chamber adaptation in athletes are the type of sport they engage in and their specific training regimen [15].

### 2.1. Cold

The interaction between cold and exercise presents unique challenges. Recent studies have shown that exercising in a moderately cold environment, as compared to a thermoneutral climate, can synergistically benefit the cardiovascular system. This environment increases the body’s tolerance to stressors and promotes cardiovascular health [16]. The underlying mechanisms for these benefits include preventing excessive core temperature rise, activating the neuro–immune–endocrine network, triggering the antioxidative system, bioenergetic and metabolic remodeling, and secreting various exerkines [16]. Additionally, peripheral vasoconstriction, which results in a rise in central venous pressure due to the translocation of blood from the peripheral circulation to central circulation, leads to increased stroke volume (SV) and a surge in VO_2_ [17].

Strenuous endurance activity generates significant heat, making it easier to maintain an optimal body temperature and performance level in moderately chilly ambient temperatures. The effect of ambient temperature on aerobic exercise performance follows a continuum; for instance, a 10 °C ambient condition is ideal for marathon competitions [18]. However, performance declines exponentially as temperatures rise beyond this optimum, although factors such as heat exchange, exercise mode, and other climatic conditions like wind, humidity, and radiation also play a role [19].

Humans possess remarkable thermoregulatory capabilities, allowing adaptation to various temperature changes, particularly in cold climates [3]. However, exposure to extremely cold environments, such as cold air or water, can disrupt proper thermoregulation, affecting the balance between metabolic heat generation and dissipation and potentially leading to hypothermia (core temperature < 35 °C or 95 °F) [20]. Consequently, exercising in very cold climates can impact performance, strain the cardiovascular system, and pose health risks [21]. For example, a 0.5–2.0 °C drop in core temperature results in an increase in resting metabolism and a reduction in Q, and peak VO_2_ [22].

When skin temperature falls below 35 °C (95 °F), heat loss from the body decreases, leading to rapid cooling, especially in exposed and distal areas such as the face, fingers, and toes. This cooling can impair motor function, manual dexterity, and tactile sensitivity. However, periodic vasodilation helps prevent vasoconstriction in these areas, potentially offering some protection against cold injury [23]. On the other hand, shivering—rhythmic contractions of skeletal muscles—can increase the body’s metabolic rate, with maximum heat generation reaching nearly 46% of VO_2_ max, or a sevenfold increase in resting metabolism [17,24].

The risk of hypothermia is significantly heightened when exposed to cold water (e.g., submersion) due to the approximately 20-fold increase in convective heat loss compared to air [21]. In such cases, the cold shock response may be triggered, characterized by an involuntary inspiratory gasp, followed by uncontrollable hyperventilation and tachycardia, along with an increased release of stress hormones [25].

Another external factor to consider is wind, which can have highly variable effects on athletes depending on wind temperature, velocity, direction, and the athlete’s movement [26]. While wind can be advantageous in some sports events, in a cold environment, it accelerates heat loss from the skin, which may not be desirable [26]. In scenarios like mountaineering at high altitudes, wind combined with wet clothing can quickly replace the air layer on the skin enriched by evaporating water with drier air, leading to further sweat evaporation and increased cooling [14,20]. Activities like running and skiing create wind across the body, contributing to windchill effects [14]. Notably, wind increases convective heat loss on exposed skin, raising the risk of frostbite, which occurs when skin and tissue are exposed to temperatures below 0 °C [14].

Finally, in events where high speeds are inherent (e.g., cycling), increased wind flow can facilitate heat release, and higher ambient temperatures can reduce air density and aerodynamic resistance. This means that high heat and humidity may not impact these sports as severely as they would slower-speed activities like athletics or beach volleyball [14].

### 2.2. Heat

When exposed to elevated outdoor temperatures and humidity, athletes are at an increased risk of excessive fluid loss and exertional heat illness [6]. Climate change may exacerbate this risk by causing more frequent and prolonged heat waves [6].

In hot weather, dissipating heat through conduction, convection, or radiation becomes more challenging, necessitating a greater reliance on evaporation for thermoregulation [27]. Sweat evaporation, the primary heat-dissipating mechanism in exercising athletes, triggers peripheral vasodilation and increased sweating. Endurance athletes, in particular, have highly efficient heat-dissipating mechanisms [20]. During exercise, most of the energy produced is converted to heat, which raises the athlete’s core body temperature [20]. This core temperature may further increase if heat-dissipating mechanisms are overwhelmed by factors such as extreme heat, humidity, dehydration, or inappropriate clothing [6,20].

The hypothalamus detects this rise in core temperature through thermodetectors, prompting sweating and increasing blood flow to the skin while reducing flow to non-essential internal areas; this shift reduces venous return [20]. Consequently, during exercise in a hot environment, HR increases to maintain Q, despite a decrease in SV [27]. However, since Q cannot increase beyond a certain point, vasodilation and blood supply to working muscles become restricted to prevent dangerously low BP [28]. If the body becomes dehydrated due to insufficient rehydration, thermoregulation is impaired, leading to decreased perspiration, reduced cutaneous blood flow, and increased heat storage. This rise in body temperature causes further HR increase, hyperventilation, disorientation, and potential cardiovascular collapse [28,29,30].

Maintaining exercise under these circumstances, with continued blood volume loss, leads to a diminished ability to dissipate heat, resulting in reduced performance, increased fatigue, and a higher rate of perceived exertion [27,31].

Humidity further complicates heat dissipation by delaying sweat evaporation and hindering the body’s ability to release heat [26]. Because high humidity and temperatures severely hamper the body’s capacity to regulate temperature, exercising and competing in intense heat—especially without proper preparation or sufficient heat acclimatization—increases the risk of heat-related illnesses and reduces athletic performance, particularly in endurance activities like running and cycling [29,32,33].

For example, athletes may experience a performance drop of approximately 2–3% due to the body’s negative feedback mechanisms, which counteract the rise in core temperature to prevent exceeding critical limits [34]. In conditions of high air temperature and water vapor pressure, soccer players have been found to cover less distance compared to standard conditions [35].

Prolonged exercise and exposure to high temperatures can lead to a broad spectrum of health problems, particularly affecting the cardiovascular, muscular, and neurological systems. These issues include exertional rhabdomyolysis, exercise-induced muscle cramps, exertional hyponatremia, prickly heat, dehydration, heat exhaustion, heat injury, and life-threatening heat stroke [6,20,36,37,38]. Exertional heat stroke, the most severe form of heat-related illness in athletes, is a medical emergency characterized by impaired thermoregulatory mechanisms and a core temperature exceeding 40.5 °C (104 °F). This condition is associated with severe mental status impairment and requires immediate and urgent external cooling measures to reduce morbidity and the risk of mortality [20]. Notably, during endurance sports in hot weather, fatal heatstroke is more common than arrhythmic mortality [39].

As mentioned earlier, the impairment of athletic performance and the risk of heat-related illnesses are more frequent in hot and humid environments than in hot and dry conditions [6,40]. Additionally, the warmer temperatures associated with climate change, especially increased humidity, have contributed to the spread of tick-borne diseases like Lyme disease in endemic areas, posing a potential concern for athletes engaged in outdoor endurance activities [6].

Finally, in contrast to cold environments, wind has a beneficial effect in hot and humid conditions [26]. Wind in such environments has been shown to result in higher power output, faster finish times, and increased physiological strain, thermal perception, and performance [26].

### 2.3. Air Pollution

Air pollution is the leading environmental factor contributing to premature deaths in Europe and is closely linked to various environmental, social, political, and economic systems [41]. Today, our environment is saturated with pollutants and synthetic substances. Over 30,000 synthetic chemicals are reportedly in use, with at least 5000 of these produced annually in quantities exceeding 100,000 tons.

The health risks posed by air pollution are comparable to those of smoking, hypertension, and inactivity, leading to both acute and long-term effects [42]. Most air pollution consists of complex aerosols, including particulate matter (PM), and gaseous pollutants, with vehicular emissions being a significant contributor.

Pollution is a well-known cause of respiratory diseases and hospitalizations, particularly among children and the elderly. Athletes are also vulnerable, as pollution can trigger exercise-induced bronchoconstriction and exacerbate asthma [43]. Beyond its impact on respiratory health, air pollution significantly affects the cardiovascular system, impairing performance and potentially leading to cardiovascular diseases [26,44].

Numerous studies and meta-analyses have shown that short-term exposure to PM and gaseous pollutants is associated with higher mortality rates. The immediate cardiovascular effects of PM may be explained by an increase in sympathetic nervous system activity, leading to changes in HR, HR variability, and electrocardiographic features [45].

In healthy individuals, inhalation of these pollutants can cause a significant rise in diastolic BP, decreased HR fluctuations, and impaired brachial artery vasodilation [46]. Furthermore, infrared spectroscopy has shown a reduction in the reoxygenation slope after cuff ischemia, indicating a constrictive microcirculatory response that decreases blood flow and potentially impairs exercise performance [46].

However, the impact of atmospheric pollution on cardiovascular health may vary depending on individual predisposition. While air pollution can cause cardiovascular damage, its clinical manifestations may be less severe in those with low cardiovascular risk [47].

Research has documented acute exacerbations of cardiovascular events, such as myocardial infarction, stroke, heart failure, and atrial fibrillation, following brief exposure to particulate air pollution [48,49]. Long-term exposure exacerbates conditions like endothelial dysfunction, BP dysregulation, peripheral thrombosis, and insulin resistance, accelerating the development of atherosclerosis [48,49].

Endurance athletes, particularly marathon runners and cyclists, are especially vulnerable to the effects of air pollution. Increased ventilation during intense training and competitions leads to higher inhalation of PM, which can impair athletic performance [50]. Studies have shown that every 10 mg/m^3^ increase in fine PM inhalation corresponds to a 1.4% drop in performance among female marathon runners [51]. Moreover, high concentrations of ozone (O_3_) combined with elevated temperatures have been shown to amplify the detrimental effects on athletic performance [52].

Additionally, exercise efficiency is reduced during short-term, maximal-intensity cycle ergometry when high levels of combustion-derived PM are inhaled. This reduction is due to delayed inflammation, which contributes to decreased performance during subsequent exercise sessions [44,51].

Gaseous pollutants also have significant negative effects on health and performance [18]. Carbon monoxide (CO), in particular, binds to hemoglobin, impairing O_2_ transport to muscles and reducing exercise capacity [26,44,53]. Indeed, elevated CO levels have been associated with increased sub-maximal HR, decreased maximal exercise time, and the onset of angina [53].

### 2.4. Altitude

The impact of altitude on cardiovascular health and athletic performance remains a subject of ongoing debate. Interestingly, individuals who permanently reside at high elevations tend to have lower mortality rates and reduced cardiovascular diseases compared to those living at lower elevations [54]. These benefits are believed to be partially due to higher levels of HDL cholesterol and lower levels of leptin, LDL cholesterol, and total cholesterol [55,56]. A recent study involving 4.2 million individuals aged from 40 to 84 found an inverse relationship between altitude and ischemic heart disease, even after adjusting for factors like sunshine, precipitation, temperature, and road distance [57].

However, altitude significantly impacts athletic performance. As altitude increases, the barometric pressure drops, leading to a reduction in air density and a decrease in environmental O_2_ partial pressure (PO_2_) [14,58,59]. For example, while PO_2_ is 159 mmHg at sea level, it decreases to 125 mmHg at 2000 m above sea level and falls to just 59 mmHg at the summit of Mount Everest [26]. The effects of this hypobaric hypoxia (low O_2_ due to decreased atmospheric pressure) vary and are often opposing, depending on the balance between reduced barometric pressure and PO_2_ [14]. The impact on exercise performance is also sport-specific, varying by the type of discipline—whether endurance-, power-, or skill-based [14].

Reduced air density at higher altitudes can be advantageous in some sports, particularly those with a low aerobic component, such as short sprints. However, reduced air density also affects projectile motion in sports like ball games, throwing, shooting, and ski jumping, potentially lowering drag and lift forces on a ball, enabling it to travel farther with less curve [26].

On the other hand, the reduction in PO_2_ negatively affects performance in athletes involved in maximal anaerobic exercises (e.g., repeated sprints) or maximal aerobic exercises (e.g., marathons) [14]. The body compensates for lower O_2_ levels by increasing HR, ventilation, and Q to supply adequate O_2_ [60]. Additionally, exposure to altitude can temporarily raise systemic BP [60]. Over time, altitude exposure leads to hemoconcentration due to diuresis and increased erythropoiesis [14], although HR remains elevated due to the decreased SV even after Q returns to baseline [59]. As a result, athletes may experience faster fatigue [60].

Without proper acclimatization, individuals exposed to moderate altitude may suffer from neurological or respiratory conditions [14,20]. Neurological issues include high-altitude headaches, acute mountain sickness, and high-altitude cerebral edema, while pulmonary problems include acute hypoxia and high-altitude pulmonary edema [14,20]. Therefore, acclimatization is essential, typically involving an increase in O_2_-carrying capacity and hemoglobin levels due to higher red blood cell production [26].

Altitude training can significantly enhance athletes’ aerobic capacity, leading to higher peak VO_2_ levels than training at lower altitudes [61]. For instance, a training cycle of three to four weeks at an altitude between 2000 m and 2500 m has been shown to improve aerobic capacity more effectively than other training regimes [61,62]. This improvement is generally attributed to higher concentrations of erythropoietin, hemoglobin, and O_2_-carrying capacity [61], although a “non-hematological mechanism” at the genetic level has also been proposed, involving the regulation of gene expression by the nuclear transcription factor HIF-1 [61].

However, training at altitude for longer than the recommendation of three to four weeks can potentially harm athletes’ cardiovascular and pulmonary functions [61]. Moreover, altitude-related leisure activities such as downhill skiing and mountain trekking have been associated with a higher risk of cardiac events or SCD [63]. The exact reasons for this increased risk are unclear, but it is hypothesized that unusual physical exertion combined with harsh environmental factors like cold and hypoxia may contribute to SCD [64]. In elderly individuals, myocardial ischemia following exercise at moderate altitude has been linked to hypoxemia, sympathetic activation, and pulmonary hypertension [65].

Additionally, a study on ultramarathon runners revealed that a significant percentage of recreational athletes exercising at altitudes between 1800 and 3200 m showed signs of exercise-induced cardiac fatigue (EICF), with notable declines in right ventricular function, especially among older participants. Interestingly, EICF did not seem to be related to the athletes’ training status, race time, or degree of dehydration [66].

Finally, it is important to consider that temperature decreases with altitude, increasing the risk of cold injuries [20]. Ultraviolet light exposure intensifies as the atmosphere thins, so climbers need to be cautious about sunburn, skin cancer, and snow blindness. Additionally, the “high-altitude desert” environment can lead to severe dehydration due to increased water losses and the difficulty of obtaining water [20].

### 2.5. Diving

The underwater environment presents unique challenges due to immersion in water and the high ambient pressure, both of which can significantly affect human health [67,68].

As divers descend, external hydrostatic pressure increases proportionally with depth, potentially leading to cold water exposure and hyperbaric hypoxia—conditions that can promote the development of gas microbubbles in the body [67].

The respiratory system’s ability to manage increased inspiratory resistance and restricted airflow becomes critical for maintaining exercise performance under these conditions. Hypoxia and lower temperatures can also induce hemodynamic stresses, including elevated hydrostatic pressure and increased venous return, leading to central translocation of plasma volume [68,69]. Initially, cold water immersion triggers parasympathetic activity, but prolonged exposure also activates the sympathetic nervous system [4,67]. This dual activation can cause vasoconstriction and increases in mean arterial pressure, pulmonary arterial pressure, and pulmonary artery wedge pressure, heightening the risk of malignant arrhythmias in susceptible divers [69].

Sustained cold exposure and cold acclimatization significantly augment sympathetic activity, leading to further vasoconstriction and a reduction in core temperature [70]. Consequently, water immersion depresses HR such that SV is elevated, and Q remains similar to what is observed in thermoneutral water. In contrast, during breath-hold diving, Q typically decreases [70].

Over time, cold environments impair normal metabolic regulation, forcing the body to increase physiological heat production to maintain normothermia. Elite divers, for example, exhibit different cardiovascular regulation during apnea compared to untrained individuals [71], showing extensive peripheral vasoconstriction to maintain O_2_ supply to vital organs under asphyxic conditions [71].

One critical risk in diving is the development of dive-related injuries, including pulmonary edema, arterial gas embolism, and decompression sickness [69,70]. Proper preparation and identifying athletes with patent foramen ovale can help mitigate these risks. Additionally, acclimatized divers may face increased risks due to sweating and vasodilatation, which can potentially reduce plasma volume and impair blood flow during exercise [70].

Interestingly, regular SCUBA diving has been shown to promote an anti-inflammatory status, providing multiple health benefits, including cardioprotection [72].

However, diving does not only occur in cold environments; it also happens in warm water, especially in tropical areas [70]. Immersion in warm water reduces the temperature gradient across the skin and impairs the body’s ability to regulate temperature, leading to a rapid rise in skin and core temperature [70].

## 3. Precautions for Individuals Practicing Sports in Adverse Environmental Conditions

Athletes who train or compete in extreme environmental conditions must adopt several preventive measures to minimize health risks and maintain optimal performance. These measures include selecting appropriate clothing, ensuring adequate hydration, following proper nutrition, engaging in habituation exercises, and implementing organizational precautions [14].

### 3.1. Clothing and Environmental Protection

Choosing the right clothing is crucial in mitigating the harmful effects of environmental factors in outdoor sports [20]. For instance, lightweight yet protective garments such as gloves, polyester caps, windbreaker jackets, and pants can prevent hypothermia and frostbite, thereby maintaining core and skin temperatures [14,73]. Humidity is another factor to consider, particularly for swimmers wearing silicon caps, as it can impair thermoregulation by trapping heat, especially in tropical regions, leading to elevated internal body temperatures and reduced overall performance [74]. Additionally, for athletes in areas prone to tick-borne illnesses, wearing long pants, long-sleeved shirts, and tall socks, coupled with the use of insect repellents, is advisable [6].

### 3.2. Pre-Cooling Strategies, Hydration, and Nutrition

In hot and humid environments, pre-cooling strategies are vital for delaying the onset of critical body temperatures, thereby preserving athletic performance and preventing heat stress [75,76]. Effective pre-cooling techniques include using ice packs, wearing ice jackets, staying in cooled rooms, taking ice baths, and consuming cold water [75,76]. Maintaining a high level of physical fitness also significantly reduces the likelihood of heat-related issues [77].

Educating athletes on proper fluid and salt intake during prolonged endurance events in the heat, as well as emergency treatment procedures, is essential [77]. Fluid–electrolyte disturbances, such as hypernatremia and hyponatremia, can cause confusion and disorientation, often due to improper fluid and electrolyte intake. For example, exertional dysnatremia is common among marathon runners who collapse, affecting approximately 30% of such cases [78]. In hot and humid environments, maintaining core temperature during exercise is crucial. Factors that hinder this process, such as humidity, dehydration, inappropriate clothing, and poor nutrition, will increase the likelihood of heat-related illness [14,20,27].

From a nutritional standpoint, athletes should ensure glycogen levels are sufficiently replenished before engaging in cold-weather endurance sports, as cold temperatures increase carbohydrate and lipid metabolism, glucose oxidation, and glycogen and lactate utilization [27]. Depleted energy stores can lead to decreased performance and reduced heat generation. Carbohydrate intake before exercise helps maintain blood glucose levels, delays glycogen depletion, prolongs endurance, and lowers HR during exercise [27].

### 3.3. Heat Acclimatization

Heat acclimatization (HA) plays a crucial role in enhancing athletes’ exercise tolerance in hot environments [19,79,80]. HA involves repeated exposure to heat, improving sweating and blood flow, reducing body temperature and cardiovascular strain, and enhancing fluid balance and cellular protection [19,79]. This process increases Q due to a rise in HR and myocardial contractility, accompanied by reduced systemic vascular resistance [28]. Regular heat exposure through training in temperatures similar to or higher than those expected during competition improves thermoregulation, facilitating faster cooling through surface vasodilation and sweating [6].

HA strategies often involve creating artificial environments, such as climate chambers, where athletes can train under conditions similar to those they will face during competitions. Typically, two-hour daily sessions over five to fourteen days are effective [6,19,79,81,82]. Additional HA methods include using saunas or heat chambers [6]. Interestingly, experimental studies have shown that pre-cooling endurance athletes with cold/ice water immersion or specialized clothing during warm-up enhances performance [83]. Therefore, event organizers should ensure that athletes have access to the most effective facilities to mitigate heat stress during competition, training, and recovery periods [14,84].

Several studies have indicated that HA improves the heart’s capacity to sustain Q and regulate BP [29]. The induction of heat shock proteins during HA provides cardiac protection, contributing to better thermal stress tolerance [85,86]. While acute heat exposure can challenge cardiovascular function, the long-term benefits of acclimatization generally outweigh these initial challenges [77].

Adaptations include increased total body water, expanded plasma volume, improved SV, reduced HR, enhanced ventricular filling and myocardial efficiency, and increased responses in cutaneous blood flow and sweating [77]. However, the extent of these adaptations varies significantly depending on exercise intensity, duration, frequency, and environmental conditions [87].

### 3.4. Cold Adaptation

For athletes engaging in cold environment activities, developing a level of adaptation through gradual and repeated cold exposure, known as habituation, is critical. Cold adaptation can significantly reduce sympathetic nerve activation, minimize cold shock reactions, lower cardiovascular responses, and enhance performance by preserving blood flow [14,21,88]. Habituation to cold also results in a more comfortable sensation and reduced and delayed variations in HR and metabolism during rest periods and outdoor exercise [17].

Recent research has sparked increased interest in cold acclimatization and its effects on brown adipose tissue (BAT) recruitment and related metabolic consequences [89,90,91]. BAT plays a crucial role in environmental adaptation by generating heat through non-shivering thermogenesis, which helps maintain body temperature in cold conditions [92]. Studies have shown that cold acclimatization enhances BAT’s oxidative capacity and activity by reducing proton leak and decreasing shivering during acute exposure, thereby making muscle thermogenesis more efficient [93]. Some research indicates that daily exposure to cold for a period of 10 days to one month can lead to a 40–45% increase in the volume of BAT capable of glucose uptake [89,90,91,94]. The distribution of BAT in specific areas of the body, such as the neck and upper chest, may influence the effectiveness of thermogenesis [94].

Ecological variables, such as temperature, have significantly shaped the physiological adaptations of populations worldwide. Heat tolerance can be linked to ancestral variations in the selection and expression of genes involved in critical physiological processes such as renal salt handling and arterial vessel tone [95]. These differences in gene expression are associated with a better ability to withstand high temperatures. Still, they are also central to the main hypotheses about the greater susceptibility of the African population to treatment-resistant hypertension and resistance to certain classes of drugs, such as angiotensin II receptor blockers [95].

### 3.5. High-Altitude and Diving Adaptation

For athletes training or competing at high altitudes, gradual acclimatization is recommended to prevent adverse cardiovascular events. Arriving at the competition site or an equivalent altitude one or two days before beginning regular training can aid in the acclimatization process. Most athletes acclimatize to moderate altitudes within two weeks [14]. Monitoring serum ferritin levels before high-altitude exposure is also crucial, as correcting iron deficiency is important to avoid impairing erythropoiesis [14].

Finally, regular diving induces a cardiac adaptive response and promotes an anti-inflammatory status in the body, thereby conferring cardioprotection and multiple health benefits [72].

### 3.6. Organizational and Environmental Considerations

Organizing outdoor competitions in adverse conditions requires careful consideration of environmental factors. Adjusting event times to earlier or later in the day can reduce heat stress, especially in endurance exercises [6]. For example, sprinting, running relays, or football are often better performed in the late afternoon, while endurance exercise in heated environments benefits from cooler temperatures in the early morning or late evening [14]. Road race start times in hot weather should also be scheduled for the late afternoon to avoid the higher heat stress of the morning sun [31]. Minimum temperatures are established for competitions performed in cold environments to reduce discomfort and ensure safety. For example, minimum competition temperatures are set at 16 °C for open water swimming and −20 °C for cross-country skiing [25].

Athletes are continuously exposed to air pollution, which poses a significant concern during outdoor competitions. Establishing monitoring programs for local pollution and pollen levels, temperature, and potential fires in the vicinity before and during competitions is advisable. If necessary, rescheduling events may be required to ensure athlete safety [6,96].

By adhering to these precautions, athletes can exercise safely and efficiently while minimizing the risk of adverse cardiovascular events and other health issues associated with extreme environmental conditions.

## 4. Conclusions

Athletes often face extreme environmental conditions, which may be exacerbated by climate change. These conditions can impact cardiovascular efficiency, performance, and overall health. Understanding the mechanisms that influence these factors is crucial for optimizing training programs, enhancing performance, and preventing cardiovascular diseases.
